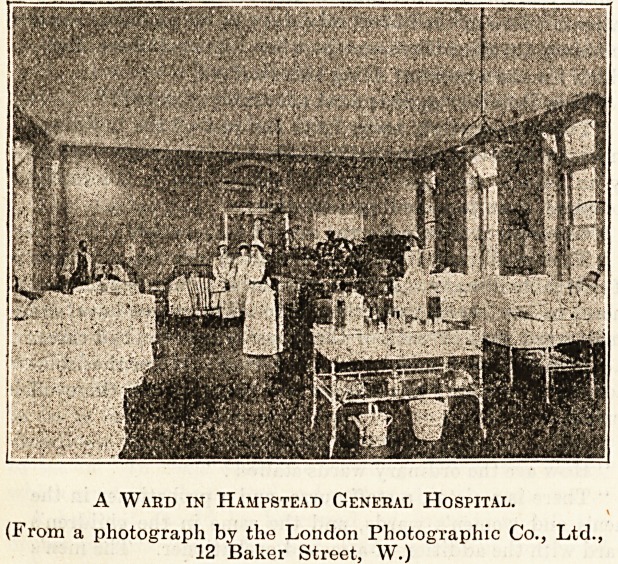# The Hospital. Nursing Section

**Published:** 1906-01-27

**Authors:** 


### The Hospital.

### Nursing Section.

Contributions for "The Hospital," should be addressed to the Editor, "The Hospital" Nursing Section, 28 & 29 Southampton Street, Strand, London, W.C.

No. 1,009.—Vol. XXXIX. SATURDAY, JANUARY 27, 1906.

### Notes on News from the Nursing World.

### RELIGIOUS INTOLERANCE AT THE QUEEN'S JUBILEE HOSPITAL.

The appointment of a new matron of the Queen's Jubilee Hospital, Earl's Court, was recently announced in our columns. Miss Fulton, matron of Noble's Hospital in the Isle of Man, who was trained at St. Bartholomew's, has undertaken a difficult task, and it remains to be seen how long she will continue to persevere with it. We are not aware whether she has read the statement made by Miss Moor, her immediate temporary predecessor, in our issue of December 2, which, as no word of denial has been forthcoming, must now be regarded as incontrovertible. In that statement it was affirmed that the only reasons alleged against her permanent appointment were that she has a certificate from the London Homœopathic Hospital and is a Roman Catholic. Apart from the fact that this must have been known when Miss Moor was first engaged as temporary matron in 1904, and was not then considered detrimental, it may be remarked that the London Homœopathic Hospital is recognised as a first-class training school, and that, therefore, an objection on the ground of her certificate could hardly be seriously put forward. As to the other allegation, it seems perfectly clear that the religion of Miss Moor was employed as an argument against her appointment. We know that a letter prejudicial to her from the religious standpoint was circulated by some one among the members of the medical staff and the Board of Management. There are other matters within our cognisance which might be referred to as showing personal breaches of good faith towards an official who admittedly deserved well of the Board. But it is sufficient just now to emphasise the irony of the situation, namely, that no official contradiction has been forthcoming to the statement that Miss Moor was mainly, if not entirely, jockeyed out of the position of matron because she professes the same faith as the Duke of Norfolk, the President of the Queen's Jubilee Hospital. We cannot believe for a moment that the Duke is acquainted with the circumstances of the case.

### UNINHABITABLE QUARTERS FOR NURSES.

Our remarks last week on the services which the inspectors under the Local Government Board are able to render to the Poor-law nurse have received fresh illustration in the report which has just been presented by Dr. Dittman following a visit to the fever house at Greenock Infirmary. In his capacity of medical inspector under the Local Government Board he points out that the provision for the accommodation of the nursing staff is exceedingly bad. There is no communication between the fever house and the other part of the Infirmary, and the assistant matron, twelve nurses, and domestic staff are thus left in a position of isolation; they are housed in a series of dingy and damp rooms, some of them in the basement of the fever house. In fact, the Inspector states that, "strictly speaking, several of the nurses' rooms are uninhabitable," and he adds that unless there are rooms available under the roof it cannot be improved. In these circumstances, it is clearly the immediate duty of the authorities to take action, and to cease to be open to the reproach that they house their nurses in quarters which are condemned as uninhabitable by an expert official of the Local Government Board.

### THE TRAINING IN SMALL SCHOOLS.

It will be seen from the interview which our Commissioner had with the matron of the Hampstead General Hospital, that Miss Gregory, while welcoming the early enlargement of the handsome new building, is by no means prepared to affirm that the training will be more valuable when the institution contains a hundred beds. She thinks that the idea of the quality of training depending upon the number of beds has been pushed too far, and that some of the most capable nurses are turned out of the smaller institutions. With regard to private nursing, her view is that the small hospitals are better. So far as Hampstead General Hospital is concerned, it certainly already possesses the equipment which is essential to admirable training, and we share the hope of the matron that a very useful future lies before it, especially as there is no idea on the part of the authorities of abandoning the practice of receiving a proportion of patients able to pay a small fee, which has been so greatly appreciated in the past.

### AN IMPOSTOR AT LARGE.

An attempt to circumvent the designs of an impostor always merits encouragement, and we rejoice that a sister at York County Hospital has had the good sense to ask us to give publicity to the story of a series of deceptions by a person whose career as a romancer cannot too soon be brought to a close. According to our correspondent's communication, this individual, whom she describes in a manner which should ensure his easy identification, has both duped hospital authorities and obtained money from hospital nurses on various plausible pretexts. Perhaps even now he would not have been found out if he had not mentioned that he had a relative who was a nurse at Winchester Hospital.

This resulted in investigations which proved him to be a fabricator of mendacious statements, and secured his ejection from York Hospital. The prominence which we are able to give to his mode of operations may be the means of rendering his admission to other institutions difficult, and of preventing any more nurses from being moved to compassion by the recital of his woes.

### GUARDIANS AND NURSES' SOCIAL GATHERINGS.

We received a few days ago a report of a social gathering and a presentation to a medical officer by the matron and nurses of Oldham Poor-law Infirmary, and we should have inserted it last week if we had not subsequently been courteously requested to withhold it on the ground that the proceedings were displeasing to the Guardians, a fact not known at the time that it was written. The meeting, however, has been mentioned in the Oldham papers, and it appears that the reason the Guardians objected to the presentation was that the relations between themselves and the medical officer in question are not of a friendly character. The answer of the nurses is that when the gathering and presentation were arranged they were in ignorance of the existence of any friction. The Guardians, however, now claim that they should in future be informed of the intention to hold social functions or make presentations. The claim may be sustained at Oldham; but bearing in mind the acknowledged difficulties experienced by Guardians generally in obtaining suitable nurses, we think that it is not good policy for them to interfere in matters which cannot be said to affect either the work or the discipline of the nursing staff.

### A NURSE'S LEGACY.

The sum of £600 has been left by the late Miss Eliot, a nurse who died at Hayward's Heath, for establishing and maintaining a cottage hospital in the town. Quite a few years ago Hayward's Heath was only a village, but it has developed so rapidly even in the present century, that it is now a town of considerable size, with the prospect of much further extension owing to its natural advantages and its proximity to Brighton. Miss Eliot has bequeathed the money for a hospital on condition that it is established by May 27, and if this is not done, the money is to be given to the Salvation Army. The townspeople, we are sure, will take care that the original intention of the testator is not frustrated, and will themselves augment the amount sufficiently to secure a building with adequate provision, and thus show how fully they appreciate the generous gift of a woman who, from her own professional experience, realised the importance to a rising town of a hospital for the benefit of its sick poor.

### THE UNCERTIFICATED MIDWIFE AT WORK.

"I should advise you to give it up, as you are not fit for it," said the coroner at an inquest at Kingsbridge, Devon, on a still-born infant child, to an uncertificated midwife. The woman said that she had acted as midwife for 16 years, and had never had a mishap before. She intended giving up nursing. The medical evidence showed that any person of ordinary knowledge should have known that the birth was not natural, and if a competent person had been present the child would probably have been born alive. The jury found that death was due to improper attendance at birth, and in giving the woman the advice quoted, the coroner told her that if anything had happened to the mother she might have been found guilty of manslaughter.

### REGISTRATION IN AUSTRALIA.

Complaints are made in Australia that nurses who hold only the certificate of a children's hospitals are placed on the general register, and it appears that in Sydney a number of these are doing general work on equal terms with fully qualified nurses. We agree with the authors of the complaints that this is an injustice to them. In England, without registration, we manage things a little better than that. Without depreciating the value of training in a children's hospital, we do not recognise it as a sufficient qualification to practise general nursing either in hospital or in private.

### A REPLY TO THE IRISH NURSES.

The protest of the Irish Nurses' Association against the proposed nursing scheme for the North City Infirmary, Dublin, to which we referred last week, has been answered by Dr. Caleb Powell, the medical officer. As to the objections to the proposed period of training, he says that nurses who are trained for three years are not usually kept for more than two in the hospital wards. With respect to the fee of 20 guineas, to which exception is taken, he affirms that "some of the hospitals exact a fee of 52 guineas," and bind the probationers to remain in their service for four years. As to the allegation that, on leaving the infirmary, nurses of inferior training will be foisted on the public, to its danger, and the discredit of the nursing profession, he characterises it in strong language, and insists that the facilities offered by the North City Infirmary for the practical teaching of nurses are exceptionally good; whilst in reply to the contention that it is unfair to receive young women for training who are ignorant of the disadvantages they would suffer later on, he declares that the nurses trained at the infirmary will always find employment in workhouse hospitals in Ireland. With regard to this declaration, however, it must be pointed out that, admitting the value of the training under the proposed scheme, it certainly would not render the nurses eligible for appointments in the general hospitals and principal Poor-law infirmaries in England, nor in the military and naval services. The Irish Nurses' Association is entitled to urge that this is a drawback which might affect the class of candidates applying for admission as probationers at the institution.

### THE IMPERIAL MILITARY NURSING SERVICE.

We are officially informed of the following changes in Queen Alexandra's Imperial Military Nursing Service:—Miss B. N. Daker, sister, has been transferred, on her return from Indian troop-ship duty, s.s. *Plassy*, to Queen Alexandra Military Hospital, Millbank; Miss M. Worthington, sister, has been transferred, on her return from Egypt, to

Colchester; Miss M. Kendall, sister, on her arrival from England, has been transferred to Wynberg, Cape Colony, and Miss K. Pearse, sister, to Standerton, Transvaal; Miss S. K. Bills, sister, has been transferred from Queen Alexandra Military Hospital, Millbank, and Miss S. Smyth, from Cambridge Hospital, Aldershot, to s.s. *Plassy;* Miss E. M. Fairchild, staff nurse, has been transferred from the Royal Victoria Hospital, Netley, to the Indian Troopship Service. Miss E. B. Darnell and Miss M. S. Williams have been appointed staff nurses provisionally.

### A PUPIL OF FLORENCE NIGHTINGALE.

BIRTHDAY greetings were recently offered to Miss Miller, who was one of the first to respond to Miss Florence Nightingale's appeal for trained women nurses. Miss Miller, who now resides in New South Wales, was eighty-two in October last, and is still in the enjoyment of excellent health. She is a native of Scotland and left her home in 1848 in order to train at St. Thomas's Hospital. Here she remained for five years and in 1868 she was one of the six nurses chosen to go out to New South Wales at the request of Sir Henry Parkes. Some days after her arrival she was selected to nurse the late Duke of Edinburgh. In 1890 she visited Scotland, but speedily returned to the Antipodes on account of the more congenial climate. She continues to take the greatest interest in the development of nursing.

### THE LONDON COUNTY COUNCIL AND PRACTISING MIDWIVES.

THE second course of lectures to midwives, which have been given under the London County Council, will finish next week. The lectures have been held in 11 centres, and 135 women put their names on the rolls at the different schools. Some of these midwives have not been able, for various causes, to attend the full course of lectures; but it is obvious that a large number have benefited, and the lectures will therefore be continued in any locality where a certain number of midwives and district monthly nurses signify their desire to attend. Miss Gill, Secretary of the Association for Promoting the Training and Supply of Midwives, Dacre House, Dean Farrar Street, Westminster, will be glad to hear from any who desire to send their names in to the Council as wishing to join the classes.

### TRIBUTES TO A NORTHAMPTON NURSE.

BY a sad coincidence two funerals closely connected with the Victoria Nurses' Home, Northampton, took place at the same hour of the day on Thursday last week. One was that of the late Mr. William Moxon, formerly Hon. Secretary of the Home, who was for many years one of its most loyal supporters. A wreath was sent from the Victoria Nurses' Home; and in addition to the Chairman of the Home, Miss Seward, Assistant Superintendent, and other members of the staff were present at the ceremony. The other funeral was that of the late Miss Gertrude Islip, a member of the nursing staff at the Home, who had recently joined it, having previously been for three years at the Northampton General Hospital. Her illness, which commenced with a poisoned finger, was contracted whilst she was helping to nurse two virulent cases of enteric fever. Both the patients recovered, and great regret is felt that the life of Miss Islip was sacrificed. The House Committee of the Home sent a floral tribute to the funeral, and others were forwarded from the Chairman, the Lady Superintendent, the nursing staff at Victoria Home, and the matron and fellow-workers of Northampton General Infirmary. The numerous mourners included Miss A. F. Lunn, Lady Superintendent of the Home, and Nurses Watts, Lowe, and Summers.

### DANCE AT LEEDS UNION INFIRMARY.

THE medical and nursing staff of Leeds Union Infirmary gave a dance in the dining-hall of the imbecile block on two evenings this month, the sisters taking charge of the decorations. The entrance hall was made very pretty with garlands and hanging baskets of sweet peas, Chinese lanterns, fairy lights, and some beautiful palms, soft red rugs covering the floor. The whole of the corridor from the male to the female side was thrown open as a promenade, and was so delightfully decorated that a long bare corridor became quite a summer bower, with plenty of comfortable resting places for the dancers. The dining hall was converted into an ideal dancing room, the floor being perfect, and the decorations representing autumn were unique and effective. The Chairman of the Infirmary Committee acted as M.C. the first night, and an ex-Guardian, who takes a great interest in the nursing staff, on the second. Dancing commenced at 8 P.M., continuing until 1 A.M. with great zest, when the National Anthem was played. Both visitors and staff thoroughly enjoyed themselves.

### SHORT ITEMS.

THE hospital at Templecombe, Somerset, built by Lady Theodora Guest in memory of the late Mr. Merthyr Guest, and known as "The Merthyr Guest Hospital," was opened this month by the Bishop of Bath and Wells. The new matron has been appointed.—Miss Hannath, the new matron and superintendent of nurses at the Wolverhampton and Staffordshire General Hospital, previous to her appointment as assistant matron at Birmingham General Hospital, acted as sister at the London Temperance Hospital, night superintendent at the Bristol Royal Infirmary, and matron at Bristol Union Hospital.—The Emperor of Japan has conferred upon Miss Lyddel, a British lady, a medal with a blue ribbon in recognition of her steady devotion since 1890 to her work among the inmates of the leper asylum in Tokio.—We are informed that the late Mrs. Elder's bequest of £50,000 is provided as an endowment fund for the Elder Cottage Hospital, Govan, not the Cottage Nurses' Training Home; but the hospital is to be used in connection with the Training Home.—The nursing staff of Strood Union Infirmary, assisted by a few friends, gave an excellent entertainment to the patients and inmates on Wednesday evening last week.

### The Nursing Outlook.

"From magnanimity, all fear above;
From nobler recompense, above applause,
Which owes to man's short outlook all its charm."

### THE CHARITABLE NURSE.

THE differences of opinion which were expressed at the meeting last week of the Royal British Nurses' Association in respect to the Registration Bill promoted by that organisation only emphasise the importance of deferring legislation until there is at any rate an approach to unity of purpose among those who claim to be the representatives of nurse-training schools or large bodies of post-graduate nurses. It cannot be pretended, either in the interests of nurses themselves or of the public, that there is any need for hurry. Nurses are far from being downtrodden or friendless, unable to secure justice or fair play. The standard of nursing has steadily improved without registration, and the position of nurses has never been so strong as it is at the present moment. As to the public, it is no exaggeration to say that there never was a time when the sick poor received so much attention, both in hospital wards and, through the agency of the district nurse, in their own homes. The rich and the well-to-do have long been able to obtain first-class nursing if they are willing to pay for it; and even the middle classes can now, by means of institutions recently started, enjoy, for a modest fee, the same advantages as the rest of the community.

If, however, registration is a question which cannot be labelled "urgent," there is a point affecting the character of nurses which imperatively demands attention. It is significant that in the United States, where registration is in operation, attention has been called to this point, and it has been suggested that the motto for 1906 of American nurses should be "Charity." The charity to which allusion is made is not, of course, the giving away of money. Neither in America, nor in England, is it requisite to enjoin upon nurses the duty of sharing their hard-earned money with the necessitous; their tendency is rather to be unduly generous. But there is another kind of charity in which it is affirmed that they are sometimes sadly deficient on the other side of the Atlantic, and we are afraid that nurses in this country are not free from a similar reproach. It is the frequent absence of charity towards each other which is lamented in America, the proneness to hasty and unkind words, to unjust judgments, to misconception of motives, to repetition of ill-natured remarks, to exaggeration of small faults or peculiarities, which do so much to cause friction in institutions. We make no comparisons, but there is no doubt that the conditions prevailing there prevail here, if, perhaps, to a more limited extent; and that their prevalence is a fruitful source of discord and discomfort. It is not insinuated that nurses are more addicted to the lack of the charity that thinketh no evil than other people, but owing to the circumscribed world in which they live, the practice of the virtue is more difficult, and the harm done by the non-practice of it is more disastrous. The disposition in institutions is naturally to talk about persons rather than things, and it is almost impossible to indulge in constant "personal" conversation without lapsing into uncharitableness unless a strong curb is kept on the tongue. Yet nowhere is the influence of kindness of speech more beneficently felt, and nowhere is it more likely to be contagious. The example of the sisters is sure to be copied by the nurses, and the newly-admitted probationers inevitably take their tone from their seniors. This involves a responsibility which is not always adequately realised; it supplies a double motive for the cultivation of charity by all who consider it to be a privilege to nurse, and do not imagine that they confer an honour upon nursing by engaging in it.

The evil of the lack of charity, which, we gladly admit, may more often be ascribed to want of thought than to any wish to inflict injury on others, lives after the nurse has left her training school. If it causes strife in the institution, it is more potent still for mischief outside. The private or district nurse who has not endeavoured as a probationer to deserve that highest of all encomiums, "I never heard her say a nasty word about anyone," will find it increasingly hard, as she proceeds on her career, to shut her eyes to the weaknesses of others, or, indeed, as time goes on, to recognise the fact that they have any pleasing traits, or can have acted disinterestedly. It is unfortunately true that the advent of a nurse is dreaded in some households, and we believe that this is in a large measure because of the existence of the uncharitable nurse. We want to get rid of the uncharitable nurse altogether. So long as she is one of the products of the training schools, there will continue to be a prejudice against nurses which the vast majority have not merited. If only every probationer could carry with her certificate of efficiency the guarantee that she possesses the milk of human kindness, she would be welcomed in homes as a help where she is now dreaded as a hindrance to the servants, and a source of irritation to the patient's friends. The charity which contributes so materially to her usefulness and is so essential to the pursuance of her vocation in the right spirit, is a plant which grows but slowly, and unless the cutting strikes root quite early in the seclusion of the training school, it cannot be expected to bloom in full beauty when it is exposed to the searching winds of every-day life.

### The Care and Nursing of the Insane.

By PERCY J. BAILY, M.B., C.M.Edin., Medical Superintendent of Hanwell Asylum.

### I.—ANATOMY AND PHYSIOLOGY.

(*Continued from page* 229.)

Human bones offer all possible varieties of shape and size, but in this connection it is usual to classify them under four heads:—

1. Flat bones, of which examples are the shoulder blade and breast bone.

2. Long bones, such as the bones of the thigh and upper arm.

FIG. 4.
VERTEBRAL COLUMN—Lateral View.
1-7, Bodies of cervical vertebræ; 8-19, bodies of dorsal vertebræ; 20-24, bodies of lumbar vertebræ; AA, spinous processes; BB, articular surfaces of transverse processes for the tuberosities of the ribs; C, articular surface of sacrum.

FIG. 5.
THE BONES OF THE THORAX.
c, clavicle; d, scapula; e, true ribs; f, false ribs; h, sternum.

FIG. 6.
THE BONES OF THE UPPER EXTREMITY.

FIG. 7.
THE BONES OF THE LOWER EXTREMITY.

3. Irregular bones, like those which form the backbone.

4. Short bones, like those of the wrist and ankle.

We may consider that the functions of the skeleton are of a purely mechanical kind. It forms the groundwork upon which the body is built and around which the soft parts, muscles, etc., are arranged. By virtue of their hardness, the bones offer an effectual protection to the organs which they cover—such as the brain, spinal cord, heart, lungs, etc.—but one of their most important functions is to form levers, which are moved by the muscles attached to them, whereby motion and locomotion are produced.

The central portion of the skeleton is the backbone, or spinal column (fig. 4). This is made up of a series of irregularly shaped bones which are superimposed one upon the other. These are called vertebræ. Each vertebra consists of an anterior portion, or body, and a posterior portion or arch. These two parts are united together in such a way as to form a ring. It is these rings, placed one above the other, which form the spinal canal. There are, in all, thirty-three vertebræ, which are distributed as follows: seven in the neck (cervical), twelve in the back (thoracic or dorsal), five in the loins (lumbar). Below these there are two bones called the sacrum and the coccyx, of which the sacrum is formed by five vertebræ fused together, and the coccyx by four similarly fused together. During the earlier years of life these sacral and coccygeal vertebræ are all distinct from one another, but at about the eighteenth year fusion occurs and the five sacral vertebræ unite to form one bone—the sacrum; while the four coccygeal vertebræ unite to form the coccyx.

The sacrum is a thick and strong wedge-shaped bone, the broad end being directed upwards, and upon it the remaining portion of the spinal column rests.

The coccyx forms a small pointed bone (in man) which is curved upwards under the sacrum.

Each vertebra is separated from the one above and below it by a thick pad or cushion of cartilage or gristle, and all are bound together by strong fibrous bands or ligaments.

The sacrum is firmly fixed between the two haunch bones to which it is bound by ligaments. These three bones and the coccyx (the latter is quite unimportant) together form the skeleton of the pelvis.

The ribs, which are attached behind to the thoracic vertebræ, are arranged in pairs, of which there are twelve. Some of the ribs are connected in front with the breast-bone, not directly, but through the intervention of a piece of cartilage (fig. 5). Each rib is a long curved bone, and every one of the upper seven pairs is connected with the sternum or breast-bone by a separate piece of cartilage—the costal cartilage. These seven pairs are, therefore, called true ribs, and each pair with the cartilage and intervening sternum forms a sort of hoop round the contents of the chest or thorax. The remaining five ribs on each side are called false ribs, because they are none of them united to the sternum at all. The upper three on each side (the eighth, ninth, and tenth pairs) are connected through a common mass of cartilage with the cartilage of the lowest true ribs—the seventh pair. The lowest two pairs (the eleventh and twelfth) are quite free at their anterior ends and are hence called the floating ribs.

The breast-bone or sternum is a flattened piece of bone which forms the anterior or front portion of the thoracic wall. It is connected with the seven pairs of true ribs as has already been indicated.

The skeleton of the trunk is, therefore, made up of the backbone, the two haunch-bones, the ribs and costal cartilages, and the breast-bone.

The skull rests upon the upper end of the spinal column. It consists of a number of bones (22) which in adult life are all, with one exception (the lower jaw), firmly and immovably fixed to one another. The bones of the face form certain cavities or holes, such as the orbits which during life contain the eyeballs, the cavities of the nose and of the mouth, while those of the head form the cavity of the skull, or brain-box.

The upper limb is suspended from the trunk by means of the shoulder girdle (fig. 6). This consists of two bones on either side, the collar bone (clavicle), and the shoulder-blade (scapula). These two bones rest upon the upper part of the skeleton of the thorax and with their fellows of the opposite side may be compared to a milkman's yoke. Each clavicle is jointed at its inner or central end to the upper part of the breast-bone and at its outer end to the scapula. The clavicles hold the shoulders apart and the width of the shoulders, therefore, depends upon the length of the clavicles. The scapula is a flattish, three-cornered bone. At its upper and outer corner there is a shallow depression to which is jointed the head of the long armbone or humerus, forming the shoulder-joint. Below the lower end of the humerus are the two bones of the forearm which are called the radius and the ulna. These two bones form, with the lower end of the humerus, the elbow-joint. The radius is the bone on the outer or thumb side of the forearm, while that on the inner or little-finger side is the ulna. Of these two bones it is the upper end of the ulna which takes the larger share in the formation of the elbow-joint, but it is the lower end of the radius which takes the greater part in the formation of the wrist-joint, at which the bones of the forearm become associated with those of the wrist. In other words, it is chiefly the ulna which carries the forearm, whereas it is the radius which carries the hand. The bones of the wrist (carpal bones) are eight in number, they are bound together by ligaments and form a mass to which are attached the five bones of the palm (metacarpal bones). At their lower end tnese are jointed to the bones of the fingers and thumb; each finger has three bones, the thumb only two, which are called phalanges.

The bones of the lower limb correspond with those of the upper limb almost exactly in number and arrangement (fig. 7). On the outer side of each haunch-bone there is a deep cup-like hollow, into which fits the rounded ball-like head of the thigh-bone or femur. In the leg (that portion of the lower limb which is between the knee and the ankle) there are two bones which differ from one another very markedly, both in size and strength. The larger of the two, the tibia or shin-bone, lies on the inner or great-toe side of the leg. The other is a long slender bone, the lower end of which forms the outer prominence of the ankle. It is called the fibula, or brooch or splint bone. In the ankle there are seven small bones (the tarsal bones) most of them more or less cube-shaped, and the bones of the sole of the foot (metatarsal bones) and of the toe correspond exactly in number with those of the palm of the hand and fingers. There is one bone in the lower limb which is not represented as a separate bone in the upper limb. This is the knee-cap or patella. It lies in front of the knee-joint which it helps to protect from injury. It is tied to the upper part of the tibia by a strong fibrous band or ligament.

*(To be continued.)*

### The Nurses' Clinic.

### THE DISTRICT NURSE AND HEART DISEASE. BY MISS M. LOANE.

A year or two ago I was called in to nurse a patient suffering from common form of heart disease which causes enormous watery swellings in the legs; the swellings had already burst and were constantly discharging, and the man was slowly but obviously dying. As a young man he had sacrificed all personal happiness in order to make a home for his widowed mother, and had continued to support her for about twenty-five years, working as long as it was possible to stand. With the ingratitude found sometimes among parents as well as among children, every effort made for him was counted and grudged, and every small alleviation possible to his state cost an amount of persuasion and bribery which it is not necessary to describe.

The first requisite in these cases is a good mattress, and every care must be taken to keep it dry; a mackintosh should be obtained if practicable, and if not, flat layers of sacking, etc., must be used. The patient should wear a flannel nightshirt and lie between blankets; the shirt and the under-blanket must be changed and dried at least every twelve hours. The legs must be protected by a cradle well covered with blankets, and as the patient is always deathly cold, a hot-water bottle should be in constant use. A two-gallon whisky jar retains the heat well, provided that it is thoroughly warmed before the boiling water is poured in, and that it is carefully wrapped up in a shawl or an old blanket.

The discharging wounds should be cautiously bathed with warm boracic, and then wrapped round with cloths wrung out in warm boracic lotion, and covered with dry, warm, bath towels. When the dressings are removed they must be soaked in cold water, then washed and boiled and ironed out smoothly. Absorbent wool, burnt when removed, would be more satisfactory as a dressing, but a shilling a day would scarcely cover the expense, and patients allowed to be in this condition are as a rule very poor. Perfect cleanliness must be maintained, if not, erysipelas is likely to set in. In some cases the swelling attacks the hands, which must then be treated and protected in the same way.

A bed-rest should be borrowed or improvised if the doctor permits one to be used, and a plentiful supply of pillows is needed. If there are not enough feather pillows, soft and cleanly ones can be made of chaff at a very small cost. The bed must be raised at the foot as the patient has a constant tendency to slip down in the bed, and it is difficult and dangerous work to keep on raising him. The blocks should be 5-inch squares of wood 3 inches in height, with a depression in the centre half an inch in depth to receive the castor. Without this depression there is always the risk of the bed slipping off the block with a sudden jerk. Another method of helping the patient maintain his position is by padding a box and placing it at the foot of the bed for the patient to press his feet against. In some cases a soft bolster may be wrapped in a sheet, passed under the patient's knees, and fastened by a bandage at each end to the head of the bed.

In cases where the patient needs support behind, and yet finds relief in leaning forwards, a bed-table should be made out of a packing case and a large soft pillow placed on the top of it.

The bedstead itself should be low to permit the patient to get on and off without great effort. Sufferers from this form of heart disease often have an overwhelming desire to get up and sit by the fire. The wish is so intense that even in strictly conducted hospitals it is often yielded to, and in the patient's own home it is practically impossible to prevent it. In all probability the patient will consent to return to bed in a few minutes' time, but if not, all that can be done is to see that the chair is placed in a safe position and the patient secured from falling forward on his face while alone, by having a broad strap or shawl fastened round the body.

The patient is especially liable to bed-sores, and every care must be taken to keep the skin dry, to stimulate it, and to vary and disperse the pressure.

In addition to suffering from a feeling of deadly cold and damp, which good nursing can to some extent relieve, the patient has to endure an intolerable sense of distress and restlessness which increase until the case must partly be regarded as a mental one. Doctors are emphatic in their warnings not to oppose or contradict the patient, but to allow him to do as he wishes. The patient's appetite is always poor, and the usual directions with regard to diet are: "Give him the best food he will take, but if he sets his mind on a frizzle or a fry, let him have it."

Patients of this class are very heavy and must always be lifted with a draw-sheet and the aid of a second person. In cases where lifting the patient even a few inches might be fatal, the draw-sheet must be changed by tacking the clean one to the soiled and drawing the latter gently away.

The patient must be washed in the same way as other severe chronic cases, but with special precautions. In some cases it is necessary to use powder under the breasts, in the groins, and between the thighs, or to place absorbent wool between the thighs, or a piece of linen spread with zinc ointment.

The friends must be warned when attending on a patient suffering from heart disease never to allow or cause any sudden change of position, never to raise him too high or place him too low, and in attending to the back to avoid turning him on one side. If the patient should faint, stimulants should not be given, the clothes round the neck should be loosened, the window opened, and a fan can be used. The attendants must be calm and quiet and try to keep the patient from all excitement, even of a pleasurable nature.

Heart disease, as known to the district nurse in poor neighbourhoods, is often an agonising complaint and generally incurable, even when the victims are mere children; but it is well within her power to provide some temporary alleviation of the pain, discomfort, and mental distress.

### To Nurses.

We invite contributions from any of our readers, and shall be glad to pay for "Notes on News from the Nursing World," "Incidents in a Nurse's Life," or for articles describing nursing experiences at home or abroad dealing with any nursing question from an original point of view, according to length. The minimum payment is 5s. Contributions on topical subjects are specially welcome. Notices of appointments, letters, entertainments, presentations, and deaths are not paid for, but we are always glad to receive them. All rejected manuscripts are returned in due course, and all payments for manuscripts used are made as early as possible after the beginning of each quarter.

### The Nurses of Hampstead General Hospital.

### INTERVIEW WITH THE MATRON. BY OUR COMMISSIONER.

Considering that the patients and staff at the Hampstead General Hospital only moved into their new quarters the first week in December, they have settled down very quickly. When I visited the hospital a few days ago in order to have a chat with Miss Rosa Gregory, the matron, there were certainly workmen in the building, but everything was none the less in excellent order; and the impression produced by the tasteful colouring, the admirable scheme of decoration, and the bright appearance of the wards is very pleasing. The story of the development of Hampstead General Hospital is extremely interesting and another extension will probably soon be commenced which, when completed, will entitle it to take its formal place among the first-class nurse-training schools.

### The Training in Small Schools.

"But I do not know," said the matron, after we had been through the whole of the hospital and had reached her charming sitting-room, "that when we get one hundred beds the nurses will be more efficiently trained than they are at present. I think that the idea of the quality of the training depending upon the number of beds has been pushed too far, and that some of the most capable nurses are turned out by the smaller institutions. Indeed, so far as private nursing is concerned, my opinion is that the smaller hospitals are better. At the same time we are naturally very glad that there is to be an enlargement. The hundred beds are badly wanted, and the hospital, when finished, will be worthy of Hampstead."

"The extension will, of course, be on the same lines as the existing building?"

"At one end of the building the extension will be simply a new wing, consisting of three wards, and at the other additions to provide further paying beds, and also an out-patient department will be made."

"You have been matron for some years?"

"For two years, and I have been here for fourteen."

### The Origin of the Hospital.

"Then you are very familiar with the history of the hospital?"

"I have practically seen its entire development. As you are doubtless aware, the originator of the institution was Dr. Heath Strange, whose portrait you saw in the Board-room. He started it twenty-four years since for the purposes of training ladies who felt that nursing was their avocation, and for providing for the requirements of a class unable to afford to pay large fees, who, though wanting hospital nursing, were unwilling to avail themselves of charity."

"When did you begin to take in free cases?"

"Twelve years ago. Prior to that date the institution was called the Home Hospital. From the outset all the nurses were fully trained, and the surgical work throughout has been splendid. One reason why the change to a free hospital was made in 1896 was the dreadful railway accident at Hampstead Heath Station, when it was so apparent that a hospital for accident cases was needed. We began by taking in accident cases free and soon received others."

### The Paying Patients.

"What was the entire number of beds in the old building?"

"Thirty-five, and out of these eighteen beds for adults and some cots were occupied by patients who paid 12s. a week. Now the position is reversed. We have two large wards of fourteen beds each, one for men and the other for women, and a third with twenty cots for children, all of which are free. But the small wards which hold nine beds are devoted to the paying patients at 12s. a week."

"And you find that there is still a demand for this accommodation?"

"The beds have all been full since we came here. We make no difference in respect to the diet. The difference is that the patients have their own medical men to attend them; and this is appreciated by both patients and doctors. The former are not supposed to stay more than six weeks, but if need arise they frequently return, which at any rate shows that they are comfortable."

### Isolation of Septic Cases.

"How large is your present nursing staff?"

"It consists of four sisters on day duty, one sister on night duty, four staff nurses fully trained, one receiving-room nurse, one theatre nurse, six first-year probationers on day duty, five in their second year on night duty, and a first year probationer who is not left in charge of any patient. Two of the nurses take duty in the isolation ward and are quite alone there, except when I go up and see them, which I make a point of doing twice a day."

"Your isolation ward is most admirably planned?"

"Yes, it is entirely away from the rest of the building, and has the advantage of being at the top. It is approached by an outside staircase from the bottom, and is absolutely complete, even to including a kitchen."

"Of course it is only for the care of your patients?"

"That is all, and not for scarlet fever nor for any cases of notifiable diseases. A case of measles would be removed there, or any septic case, though in addition we have one small ward shut off each of the larger wards for bad cases. A dying patient would be removed here. When the isolation ward is empty the nurses take the place of those off duty."

### Age and Salary.

"How are the ordinary wards staffed?"

"There is a sister, a staff nurse, and a probationer in the men's and women's wards, and the same in the children's ward with the addition of a second probationer. The men's ward is called the Albert Victor, after the son of the Prince and Princess Christian, who died in South Africa."

"At what age do you admit probationers?"

"Between twenty-two and thirty. I have always plenty of applications, notwithstanding that payment of a deposit of four guineas is required. Some years ago the amount was ten guineas, and it was occasionally found too high. The deposit is returned at the end of the first year, or if a probationer has to leave from any cause within three months she gets it back. No salary is paid the first year, but £12 in the second, £20 in the third, and £25 the fourth; or, if the nurse is serving on the private nursing staff £30 and 5 per cent. commission on earnings."

### Off-duty Time.

"When did you increase the term of training to four years?"

"In preparation for our removal here. We provide indoor and outdoor uniform. I am sure that the latter is appreciated because it saves time when a nurse has only a couple of hours to go out. No nurse is required to wear it on her day off duty. As to the hours of duty, the day nurses are in the wards at 7.30 A.M. and leave at 8.30 P.M., with half-hour intervals for dinner and tea. In addition to the two hours daily off-duty,

### THE NURSES OF HAMPSTEAD GENERAL HOSPITAL—*continued.*

half an hour is allowed after the morning's work. The night nurses are on duty from 8.45 P.M. to 8.30 A.M., and their off-duty time varies; from November to March it is from 9 to 11 P.M., and from April to October, when they rise earlier and go to bed earlier, from 5.30 to 8 P.M. Every nurse has a day off once a month and three weeks' holiday during the year."

### The Private Staff.

"Have you had a private nursing staff for many years?"

"There has been one from the first, and generally trained here. Just now the number is small, because we have wanted so many for the hospital. We shall get more as we train more. They are always out. The private nurses live at our institute in Pond Street, and the night nurses attached to the hospital sleep there, a sister being in charge."

### The Lectures.

"By whom are the lectures given?"

"By the visiting staff. We have only one resident medical officer. The matron or sisters instruct the probationers in their first year in the details of practical nursing, and they also attend elementary lectures on anatomy, physiology, and first aid. In the second and third year, when the probationers are trained nurses, they attend lectures on the principle and practice of nursing and on such knowledge of medical and surgical diseases as may be deemed desirable. A final examination is held on these subjects and also in anatomy and physiology at the end of the third year. There being no school here the nurses are practically dressers."

A Ward in Hampstead General Hospital.

(From a photograph by the London Photographic Co., Ltd., 12 Baker Street, W.)

"Is there any instruction in cooking?"

"The nurses have just been attending a series of lectures on sick-room cookery at the Northern Polytechnic. I think that a knowledge of cookery is very important, especially for private nurses who ought to be able to see that cooking is well done, and who sometimes have to do it themselves."

### The Nurses' Quarters.

"Except the night nurses, the staff are housed in the hospital?"

"Yes. As you have seen, their quarters are quite separate. Each nurse has a bedroom to herself with a fire-place, and a fire is lighted once a week, or in case of illness. There are no radiators in the rooms. Two bathrooms are provided on each floor, there are separate sitting-rooms for the sisters and for the nurses, and a dining-room for the staff. We have had a grand piano given us, but we have no space for it until the other wing is built. I pointed out just now the intended nurses' sitting-room and some of the bed-rooms for future nurses are for the same reason used for patients."

"How is it that you have no room for the sister leading off the wards?"

"I hope that will be remedied when the hospital is completed, and each sister is in charge of two wards. The staff will then have to be increased by about a third. It seems rather large just now, but we could not do with fewer nurses and allow the same time off duty. We make it a point not to interfere with that."

### The Practice of Economy.

"The out-patient department," continued the matron, "is at present in Pond Street, with a nurse in charge, and we have no laundry. But I hope to have the latter shortly. It means a great saving of expense. I have no housekeeper, but only a linen-room maid, who manages the stores. Some people say that we pamper our patients, but as the food average per head in 1905 was only 6s. 8d., I do not consider that for a small institution, we can be justly accused of extravagance. The sisters have purposely been selected from several of the different leading schools, and we hope that as a training school there is a very useful future lying before the Hampstead General Hospital."

### The Registration of Nurses.

### ROYAL BRITISH NURSES' ASSOCIATION.

A special general meeting of the Royal British Nurses' Association was held on Wednesday afternoon last week to consider the redrafted Bill of the Association for the State Registration of nurses. Dr. Bezley Thorne presided, and there was a large attendance of members. The clauses of the Bill were read, and, with the exception of certain clauses relating to the constitution of the Central Board, were approved.

Miss Forrest, of Bournemouth, moved the deletion of the three last sections of Clause 4 and the restoration of certain sections of the original Bill providing for the appointment of five (instead of four) fully-trained nurses, who should be Matrons or Lady Superintendents of hospitals or Poor-law infirmaries with training schools attached, and seven fully-trained nurses.

Dr. Berkeley pointed out that this would materially increase the number of members of the Central Board, and the Select Committee of the House of Commons had objected that the number previously proposed was too great.

Miss Forrest replied that in those circumstances the number of medical practitioners whom it was proposed to elect should be reduced.

Miss Helen Todd, Matron of the National Sanatorium for Consumptives at Bournemouth, said that it would be as great an anachronism to have 80,000 nurses represented by medical men as it would be to have the Royal College of Physicians represented by surgeons and the Royal College of Surgeons represented by physicians. Nurses ought to be represented by women who knew their needs.

Mrs. Bedford Fenwick said that the nurses of the kingdom wanted full and sufficient direct representation, and that was not provided for in the Bill. They would not allow the great body of nurses throughout the country to be enslaved by a little community such as the Royal British Nurses' Association. Whatever they passed would be defeated if it was injurious to the future profession of nursing. They had confidence in the Government which was coming into power, and they knew that their position would receive consideration.

The Chairman said that if the proposal were carried membership of the Central Board would be increased to 21, whereas it was the strongly-expressed opinion of the Select Committee that it should not exceed 15.

The amendment was lost.

Subsequently Clause 11 was altered so as to read "one registered nurse to represent London, one to represent Scotland, one to represent Ireland, and two to represent Wales and the provinces."

It was further provided that the representative to be appointed by the Association should be a nurse.

The rules defining the duties and powers of the Central Board were afterwards discussed.

### SOCIETY FOR THE STATE REGISTRATION OF NURSES.

On Friday last a meeting of the Society for the State Registration of Nurses was held at 20 Hanover Square, to consider the re-drafted Bill for the State Registration of Trained Nurses promoted by the Society. The Matron of St. Bartholomew's Hospital. Miss Isla Stewart, presided. The Bill, which makes provision for the representation of the registered nurses on their governing body, was adopted.

Miss Helen Todd, Matron of the National Sanatorium for Consumptives at Bournemouth, proposed, and Miss Mary Burr, member of the Royal British Nurses' Association, seconded, the following resolutions, which were unanimously agreed to:

"That this meeting most strongly condemns the retrograde action of the Executive Committee of the Royal British Nurses' Association in eliminating from its redrafted Bill for the State Registration of Trained Nurses almost the whole of the direct representation on the Central Board originally accorded to trained nurses."

"This meeting further considers that any Registration Bill which does not make full provision for the direct representation of trained nurses on their governing body contravenes a cardinal principle of justice, and should be actively opposed."

### Everybody's Opinion.

### AN EXPERIENCE IN EDINBURGH.

Isabella Brown, District Nurses' Home, 3 Buccleuch Place, Edinburgh, writes: Will you kindly allow me to acknowledge with many thanks in The Hospital the sum of 5s. which I have received for the "old body" in the article headed "An Experience in Edinburgh." No name was given me, but the "old body" was grateful for the gift kindly sent her.

### PRIZES FOR THE FINEST BABIES.

The Secretary of the Acton Nursing Institute, Massage, Midwifery and Maternity Training School, writes: Whilst thanking you for the notice you were kind enough to give us in last week's Hospital, I beg to call your attention to a slight error which might create a mistaken impression among your numerous readers. The tea and entertainment given on the 8th inst. were the first of three portions, and out of 130 invited nearly 80 turned up of the first portion. During the past twelve months upwards of 450 patients have been attended by our nurses, therefore, as the notice reads, it gives people the idea that we have had only 70 patients during that period.

### MENTAL TRAINING FOR POOR-LAW NURSES.

The Medical Officer of Willesden Workhouse and Infirmary writes: The note appearing in your issue of January 20 headed "Mental Training for Poor-law Nurses" contains a misleading report of the inquest held at the Middlesex Asylum on the previous Tuesday. At the inquest no nurse gave evidence; an attendant of the workhouse did so, however, and this witness spoke of the condition of the patient at the time of her removal as "exhausted," but certainly did not state "that she was in a dying condition." However, the main defects of the report are in the account given of my own evidence, and especially in the omission of the most important part of it. The facts of the case shortly are as follow: The patient was admitted to the workhouse suffering from mania—no doubt consequent upon broncho-pneumonia (probably influenzal), for which she had been treated for some time outside. Feeding by the nasal or stomach tube—a procedure which you are aware may not be undertaken by a nurse—was the only means by which nourishment could be given. Her condition on Tuesday, January 9, was such that she required feeding at frequent intervals, and hence as there is no medical officer resident at the infirmary I had her removed to the asylum, where she died two days later. At the inquest the jury, as you point out, exonerated the officials concerned, but nevertheless in consequence of the omission above referred to, by speaking of the case as broncho-pneumonia, and in other respects—the report given clearly permits an inference to be drawn detrimental to the ability and competence of the trained nursing at the Willesden Infirmary. Such a result I am assured you would be the last to desire, and I thank you in anticipation of your kindly inserting this letter in your next issue. I need hardly say that I am in entire agreement with your remark as to the advantages of an infirmary staff including nurses who have had mental training.

[Our report was necessarily only a summary, but we are glad that Dr. Webster agrees with us as to the advantage of an infirmary staff including nurses who have had mental training. The object of our note was to point out this advantage, and we do not think that it suggested any doubt as to the ability or competence of the trained nurses at Willesden Infirmary.—Ed. The Hospital.]

### A WARNING TO NURSES.

"Sister B.," County Hospital, York, writes: Will you kindly allow me space to warn nurses against an impostor? His mode of operations is to come up to a hospital in the evening, between 8 and 9 p.m. as a rule. He asks to see the house doctor, and represents himself as ill and "down on his luck." His temperature is between 101° and 102°. His disease varies, but is usually, I believe, Tertian malaria. He says that he is a gentleman, and his occupation, like his disease and his address, is not always the same. He has represented himself as a clerk, a singer, and a doctor, and if in the north he gives an address in the south, or vice versa. When admitted he proceeds to make himself most fascinating to the nurses and sister of the ward. He tells heartrending tales of himself and his unkind stepfather, and he probably makes violent love to the most tender-hearted and innocent of his nurses, and by this means gets as much money as possible before he is discharged. He is tall, about 6 ft., rather good-looking, medium coloured hair and complexion—*i.e.*, he is neither dark nor fair, but rather fair than dark—irregular teeth, several missing at the sides in the top jaw; rather prominent jaw; eyes light grey or blue-grey; clean shaven; hands long and thin; age about 30 to 33. He has a faint scar about 1 in. or 1½ in. long on his left temple, above and parallel with the end of the eyebrow. But this is so faint that it probably would not be noticed unless carefully looked for. He is tatooed on both forearms. Unfortunately, the designs were not carefully noticed, but they are common ones, including clasped hands, and crossed flags. The first time we know of his getting into a hospital is at Winchester on September 21, 1903. He gave his name as Treville, and remained there till October 13. On leaving, money was given him from the Samaritan Fund. He was in Derby Infirmary between October, 1903, and May, 1904—I think in March—and got £5 from one of the staff on leaving. He appeared again at Winchester on May 30, 1904, and bolted on June 12. He had written to Derby to try and get some more money from the same nurse, and she wrote to Winchester to make inquiries about him. Unfortunately, she wrote to him by the same post saying what she had done, and he vanished as soon as he got the letter. He was admitted here on December 15, 1905, suffering from "Tertian malaria." He gave his name as Hudson Marsden, M.R.C.S., L.R.C.P.Edin., and late house surgeon at the Devon and Exeter Hospital. Luckily, he said that he had a sister nursing at Winchester Hospital, and inquiries were made, resulting in the finding out of the facts I have set forth above. The sister, of course, was non-existent. Being charged with his lies, he at first denied and then admitted everything, and, of course, went out at once. Judging from his knowledge of the Devon and Exeter, I should say he had been a patient there while the real Mr. Marsden was hospital surgeon. I believe he was also at Leicester, posing as an "honourable," and got a collection from the staff. But this is not certain. I hope this letter may help to check his successful career.

### Appointments.

Children's Hospital, Bradford.—Miss Beatrice Smith has been appointed sister. She was trained at North Riding Infirmary, Middlesbrough, and has since been staff nurse at the Royal Infirmary, Derby, and sister at the General Infirmary, Burton-on-Trent.

Infant Orphan Asylum, Liverpool.—Miss Florence Coker has been appointed matron. She was trained at St. Thomas's Hospital, London, and has since been home sister at the Yarrow Home, Broadstairs, and house matron at the Downs School, Sutton, Surrey.

Merthyr Guest Hospital, Templecombe, Somerset.—Miss Sarah Bardwell has been appointed matron. She was trained at the Salisbury Infirmary, and has for some years been connected with the Nurses' Home.

Miller Hospital and Royal Kent Dispensary, Greenwich.—Miss Rosa C. B. Watts has been appointed matron. She was trained at Poplar and Stepney Sick Asylum, where she was afterwards staff nurse, ward and theatre sister. She has since been night superintendent at the Blackburn and East Lancashire Infirmary, and superintendent nurse at Bethnal Green Infirmary. She has also done matron's duty at Croydon Poor-law Infirmary, and has had experience in private and district nursing. She holds the certificate of the Central Midwives Board.

Montagu Hospital, Mexboro', Yorkshire.—Miss E. Hughes has been appointed staff nurse. She was trained at the Hope Hospital, Pendleton, Manchester, and has since been staff nurse at Birkdale Hospital.

Queen Victoria's Jubilee Institute for Nurses, Barry, South Wales.—Miss Agnes Tyson has been appointed superintendent. She was trained at the North Staffordshire Infirmary, Stoke-on-Trent, and for two years worked as Queen's district nurse in Hull. She has since been matron of the Ulverston Cottage Hospital, and has subsequently done private nursing in connection with Grosvenor House, Southampton.

Royal Victoria Hospital, Bournemouth.—Miss Marion Kate Watkins has been appointed charge nurse. She was trained at the Royal Infirmary, Windsor, and has since been charge nurse at the Royal Hospital, Richmond, Surrey, and staff nurse at the Hospital for Women, Soho Square, London.

St. Luke's Hospital, Halifax.—Miss M. A. Barnes has been appointed sister. She was trained at St. Luke's Hospital, Halifax, and has since done private nursing at Nottingham. She also helped during the epidemic of typhoid fever at Lincoln. She holds the certificate of the Central Midwives Board.

Southwark Infirmary, East Dulwich Grove.—Miss Beatrice Nye has been appointed night superintendent. She was trained at Luton Hospital and Charing Cross Hospital, London, where she was afterwards staff nurse. She has since been sister at the Hospital for Women, Liverpool.

The Downs School, Sutton, Surrey.—Miss M. E. Y. Wood has been appointed house matron. She was trained at the Royal Free Hospital, London, and has since been night superintendent at Croydon Poor-law Infirmary and nurse-matron at the Probationary Blocks of the Shirley Schools, near Croydon.

### Queen Victoria's Jubilee Institute for Nurses.

Her Majesty Queen Alexandra has been graciously pleased to approve the appointment of the following to be Queen's Nurses, to date January 1, 1906.

ENGLAND.

| Name | District Training at | Serving at |
|---|---|---|
| Elsie Annie Mary Friend | Bermondsey | Trumpington |
| Ada Dicks | Bloomsbury | Gillingham |
| Winifred Alice Giles | „ | Sandown |
| Agnes Mary Ogle | „ | Penzance |
| Jessica Beatrice May | Brighton | St. Austell |
| Violet Muriel Pearson | „ | Hove, Brighton |
| Helena Margaret Gelder Brook | Camberwell | Bath |
| Emma Ann Pasfield | Cardiff | Northampton |
| Lucy Maud Hawkins | East London (Cable Street) | East London |
| Maud Gwendolyn Hay | „ | „ |
| Ena Barton | East London (Stepney Green Div.) | „ |
| Laura Helena Chapman | „ | „ |
| Annie Cordelia Bevan | Gateshead | Gateshead |
| Miriam Dagmar Cole | Gloucester | Portsmouth |
| Elizabeth Emens | Hull | Hull |
| Henriette Marie Bondix | Kensington | Kensington |
| Minnie Adelaide Jarvis | Kingston-on-Thames | Kingston-on-Thames |
| Emily Ada Bentley | Leeds (Holbeck Home) | Leeds |
| Esther Elizabeth Littler | Liverpool (Derby Lane) | Liverpool |
| Fannie Amelia Jones | Liverpool (North Home) | „ |
| Jeanie Skimming | „ | „ |
| Blanche Fiddes | Liverpool (Shaw Street) | „ |
| Agnes Pool | „ | „ |
| Georgie Simpson | „ | Huddersfield |
| Lucy Hudson | Manchester (Harpurhey) | Manchester (Harpurhey) |
| Jane Simpson | „ | „ |
| Christena Anderson | Portsmouth | Accrington |
| Mary Anne Carter | „ | Carlisle |
| Catherine Anna Paling | „ | Grimsby |
| Edith Annie Jennings | Salford | Manchester (Hulme Home) |
| Clara Jane White | Sheffield | Sheffield |
| Emily Myfanwy James | Shoreditch | Gloucester |
| Rose Anne Wood | „ | Hayle, Cornwall |
| Annie Packe | Northants D.N.A. | Clipston, Northants |
| Mabel Caroline Frances Arnold | Hammersmith | Kensington |

WALES.

| Name | District Training at | Serving at |
|---|---|---|
| Mary Elizabeth Stevenson | Cardiff | Treorchy |

IRELAND.

| Name | District Training at | Serving at |
|---|---|---|
| Maggie Early | St. Lawrence's, Dublin | Dublin |
| Josephine Mary Jackson | „ | „ |
| Susan Josephine McQuaid | „ | Mount Talbot |
| Jane Hall | St. Patrick's, Dublin | Dublin |

SCOTLAND.

| Name | District Training at | Serving at |
|---|---|---|
| Katherine Boyd Cameron | Scottish District Training Home, Edinburgh | Lochwinnoch |
| Helen Grant | „ | Edinburgh |
| Annie Hardie | „ | Alloa |
| Helen Jean McAlpin | „ | Edinburgh |
| Sara McGifford | „ | Tollcross |
| Ethelwyn Mary Narracott | „ | Gourock |
| Agnes Reid | „ | Musselburgh |
| Alice Maud Rennie | „ | Rosewell |
| Mary Webster Spence | „ | Kirriemuir |
| Kathleen Maria Steele | „ | Wishaw |
| Rachel Alexander Thorburn | „ | Johnstone |
| Cecilia Watson | „ | Crieff |
| Janie Reid McHardy | Aberdeen | Aberdeen |

### A Book and its Story.

### A SCOTCH HEROINE.*

THE suggestion of a new book by a new writer has an attraction for most readers, and in the one before us, happily named "A Heart's Harmony," there are many things that not only attract but hold the attention of the reader from start to finish. The characterisation is excellent; and pathos and humour are delicately blended in Hester Campbell, the heroine, a clever and artistic study. Whether as a child when left motherless in charge of a faithful Scotch servant, Elspeth, a delightful specimen of her class, and an absent-minded scholarly father, she charms us by her originality, or as from girlhood to womanhood we follow her through the sorrows that shadow but cannot daunt the brave heart that finds relief in devotion to duty and a life lived for others, she is always natural and swayed by sweet reasonableness. Her character is a dominant one. Thoughtful and self-reliant, with an imaginative temperament and much intellectuality, she leads a dual life. There is the busy helpful one which is claimed by her devotion to her father and the people under his charge, and the life of visions into which music and books, in her scarce intervals of leisure, uplift and transport her. Her faculty of "make believe" never deserts her, nor her sympathy with children. The charming picture of her childhood, and the scenes in girlhood when, with children around her, she spins fascinating stories on the spur of the moment, shows her, as always, spontaneously original. Three times in her life the shadow of death comes near to her. First by the death of her mother, followed by that of her only brother, to whom she is deeply attached, from an accident, and then the failing health of her father ending suddenly in heart-failure leaves her alone in the world. The following passage shows Hester at the time her father has had a seizure in the pulpit and a second opinion has been called in to report on his case. Dr. Barrett has gone upstairs to see the patient, and returns to find Hester, exhausted by a sleepless night, has fallen asleep in her sitting-room.

"He moved briskly forward, then came to an abrupt standstill as his glance lighted on the sleeping girl by the fireside. The careless grace of the slender form held his eye. With one foot curled beneath her, and the other hanging limp, its little shapely slipper scarce touching the ground, the girl slept in the easy abandonment of physical unconsciousness. The dark head drooped sideways against the chair-ledge, and a hand, palm uppermost, lay outstretched on her knee. The man's eye wandered with awakened curiosity over the room. It regarded in turn the long line of low book-casing with well-ordered array of literature running the length of one of its walls, and the worn but spotless carpet. His eye travelled to the blue spode ware on the mantel, and hanging above it a mezzotint of the Madonna and child. On the table a tray with delicate old china and a kettle stood ready for use, and the soft light of the lamp fell on a bowl of chrysanthemums whose bitter-fresh fragrance stole out to meet him. It was a well-used, well-worn room, in spite of its polished wood and subtle air of refinement. . . 'Home!' He dwelt on the word to himself, 'Home! that's the meaning of it.' Hester's opening eyes fall on the tall stranger as she struggles back to consciousness and realises his identity. Coming forward with the absence of self-consciousness which was characteristic of her, she interrogates: 'Dr. Barrett?' holding out her hand. The grey eyes meet his with unwavering regard as he apologises for startling her. 'It is not your fault that you caught me napping at this unseemly hour. I sat up part of the night with my father and have been possessed of sleep all day, and the worst of it is that it's the height of my ambition to nurse!' The man leaned forward smiling at her dismay. 'It's all a matter of use and wont. The steel is not tempered in the first fire.' Hester asks Dr. Barrett to tell her what he exactly thinks of her father. 'Everything, please. I have been used to facing things ever since I was quite young.' The dignity and frankness of the girl were a new combination in the man's knowledge of her sex. . . . The girl's eyes searched him. 'You think he can do his work, then?' 'Certainly, in measure.' Hester took the tea-things from Elspeth's hands, daintily cut bread and new baked scones. Her face lost its tense expression." Before Dr. Barrett takes his leave, in reply to a remark from Hester in which the name of her brother had come, he asks: "'You have a brother?' The words were scarcely across his lips ere he wished them unsaid. A spasm swept the mobile mouth and the grey eyes looked out of a mist of tears: after all the years Hester could not speak of her boy unmoved. 'It's many years since he died,' she said, gazing into the fire. 'You must forgive me,' the man breathed softly. As a shaft of sunshine breaking through the midst of rain, the girl's smile dispelled all thoughts of gloom. 'There is nothing to forgive. No, our household consists only of my father, Elspeth, and me, the triple cord not easily broken,' she added. . . . 'Elspeth's a dear. She's been with us ever since I was born.' 'One of the few remaining of the grand old type of Scotch servant?' 'Yes, Providence does not grow them nowadays.' 'And you, don't you ever feel lonely sitting here all by yourself?' he asked, wondering at his own temerity. A little rill of laughter ran out towards him. 'I believe after to-night you will always think of me as a forlorn young person, slumbering in solitude with my hands in my lap. I haven't time to be lonely. I'm the daughter of an absent-minded minister. I'm his incarnate notebook and calendar. I'm one of the stoops of his kirk, I'm his housekeeper and district visitor and amanuensis. There are twelve hours in a day in which a woman may work, but they're all too few for what I want to do.' The girl rose and Barrett surveyed the slight figure drawn to its full height, wondering within himself at the burden carried by such young shoulders." There are many good scenes from the lives of the poorer folk whom Hester seeks out, and the gloom of their surroundings is relieved by the humour which springs up as her invariable attendant. The setting of the story, the dialect in parts of it, and the customs are essentially Scotch. To English ideas, the incident of a wedding party arriving to be married at eight o'clock in the evening and remaining there until the absent-minded minister returned at ten, having forgotten the fact that they were due, and forthwith proceeding to make them man and wife, seems an unceremonius proceeding. Two answers to Hester's queries are worthy to be quoted. The little district nurse is asked what carries her so unfalteringly through her duties: "Two parts love, one part resolution, the fourth part humour," is the reply. When the Bath-chair man is questioned as to whether he did not find human nature pretty much the same in all ranks: "Well, I don't know as how I'd go so far as to say that. I could tell you of a Harmy officer as could swear powerful strong, hall the same 'e did the generous by me. Or I could tell you of a helderly female party, miss, as spoke to me for my good, and gave me tracts, but she stuck to 'er plain fare, she did." "A Heart's Harmony" can be cordially recommended for the admirable qualities mentioned above and for the pretty romance running through it.

* "A Heart's Harmony." By Ethel M. Forbes. (Andrew Melrose, 6s.)

### Notes and Queries.

**REGULATIONS.**

**The Editor is always willing to answer in this column, without any fee, all reasonable questions, as soon as possible.**

**But the following rules must be carefully observed.**

1. **Every communication must be accompanied by the name and address of the writer.**
2. **The question must always bear upon nursing, directly or indirectly.**

**If an answer is required by letter a fee of half-a-crown must be enclosed with the note containing the inquiry.**

*Hypodermic Syringes.*

(126) Will you advise me as to the best method of keeping hypodermic syringes ready for immediate use. Up till now I have been in the habit of soaking them weekly in olive oil, and then drawing them through strong soda water and ending with ether. I have tried keeping them immersed in absolute alcohol, but find it unsuitable for the syringes, though very satisfactory as regards the needles.—*Emergency.*

You might try carbolised glycerine.

*Army Nursing Reserve.*

(127) Can a nurse having had general training at a Poor-law infirmary get into the Army Nursing Reserve, or must she have had hospital training?—*Gamp.*

Candidates having had Poor-law infirmary training are eligible for the Army Nursing Reserve provided that they have had good surgical experience and are otherwise suitable.

*Fee.*

(128) I was engaged by a lady to nurse her niece. The lady was going to stay with her until I was wanted, and then send for me. I refused a case for that month and was never asked to go. The lady who engaged me died last week. Could I recover from her executor?—*Nurse P.*

If you have the engagement in writing, or can produce satisfactory evidence that the deceased made the engagement with you, you should decidedly be able to do so.

*Convalescent Homes.*

(129). I want to train for nursing, but as I am not old enough I have been wondering if they take probationers into Convalescent Homes. I thought it would give me an insight into a few things such as bandaging, etc. If there are any Convalescent Homes such as I mention, would you kindly name one or two?—*L. C.*

Try the Invalid Children's Convalescent Nursing Home, Winifred House, Wray Crescent, Tollington Park, N.; also the Home for Incurable Children, North Court, Hampstead, and the Home for Invalid Children, 70 Montpelier Road, Brighton.

*Training.*

(130) Would you kindly inform me which are the best Children's Hospitals, at what age probationers are received, and are salaries given?—*A. B. C.*

Consult "How to Become a Nurse," published by the Scientific Press, Limited, 28 and 29 Southampton Street, Strand, London, W.C.

*Milk.*

(131) Will you kindly inform me whether milk which is brought rapidly to boiling point has all the ordinary disease germs destroyed while its quality is not deteriorated?—*Sister Nancy.*

Its quality is, to a slight extent, deteriorated, although disease germs are practically all destroyed, by boiling the milk.

*Infant's Food.*

(132) I am about to take a baby of three and a half months old on a six weeks' voyage. There is a great controversy as to food, the baby having been brought up on the Soxhlet system of sterilized milk. One doctor recommends the Walker-Gordon milk. Can you tell me if this is preferable to putting the baby on Nestlé's unsweetened condensed milk?—*C. L. A.*

It is a matter for the medical attendant to decide.

**Handbooks for Nurses.**

| | Post Free. |
|---|---|
| "A Handbook for Nurses." (Dr. J. K. Watson.) ... | 5s. 4d. |
| "Nurses' Pronouncing Dictionary of Medical Terms" | 2s. 0d. |
| "Art of Massage." (Creighton Hale.) ... ... ... | 6s. 0d. |
| "Surgical Bandaging and Dressings." (Johnson Smith.) ... ... ... ... ... ... ... | 2s. 0d. |
| "Hints on Tropical Fevers." (Sister Pollard.) ... | 1s. 8d. |

Of all booksellers or of The Scientific Press, Limited, 28 & 29 Southampton Street, Strand, London, W.C.

### For Reading to the Sick.

GOD'S FLOCK.

Blessed that flock safe penned in Paradise;
Blessed this flock which tramps in weary ways;
All form one flock, God's flock; all yield Him praise
By joy or pain, still tending toward the prize.
Joy speaks in praises there, and sings and flies
Where no night is, exulting all its days;
Here, pain finds solace, for, behold, it prays;
In both love lives the life that never dies.
Here life is the beginning of our death,
And death the starting-point whence life ensues;
Surely our life is death, our death is life:
Nor need we lay to heart our peace or strife,
But calm in faith and patience breathe the breath
God gave, to take again when He shall choose.

*Christina Rossetti.*

It is a strengthening, calming consideration that we are in the midst of an invisible world of energetic and glorious life, a world of spiritual beings, than whom we have been "made for a little while lower." Blessed be God for the knowledge of a world like this, for we believe the wrongs of the ages will be righted there.

*Archdeacon Basil Wilberforce.*

In the highest regions there is no passive calm, no heedless indifference as of those who, being exempt from sorrow, cannot feel for those who weep. The words which are spoken by the angels to Mary contradict the idea that a higher life is a passionless life. If there is joy in the presence of the angels over one sinner that repenteth, there is sympathy also for those who weep. To this sympathy the angels give expression, "Woman, why weepest thou?" And besides the sympathy, do we not catch an undertone of kindly wonder—why should she weep? When all is known, the causes for tears are less than we imagine. In Mary's view all was lost; for Christ was dead. In the angels' view all was won, for Christ had risen. What different standpoints are expressed in the question and the answer. . . . She had as yet no perception of the victory of life over death. *Bishop Boyd Carpenter.*

The Scriptures say that the holy and just go into the unseen world, and there enjoy the most pleasant peace and sweetest rest. As in this life they were wont to fall softly asleep in the guard and keeping of God and of the dear angels, without fear of harm, though the devils might prowl about them—so, after this life, they repose in the hand of God.

When my soul departs, I know that highest kings and princes are appointed to attend me; namely, the dear angels themselves, who will receive me and guard me on my way.

*Luther.*

Angels! sing on, your faithful watches keeping,
Sing us sweet fragments of the songs above;
Till morning's joy shall end the night of weeping,
And life's long shadows break in cloudless love.
Angels of Jesus, angels of light,
Singing to welcome the pilgrims of the night!

*F. W. Faber.*

## Figures and Tables

**Fig. 4. f1:**
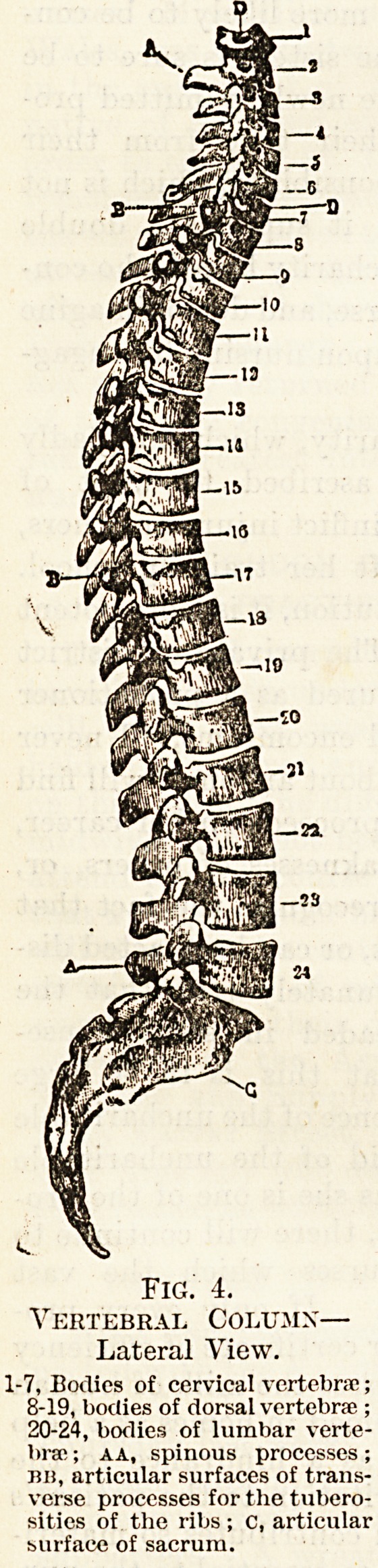


**Fig. 5. Fig. 6. f2:**
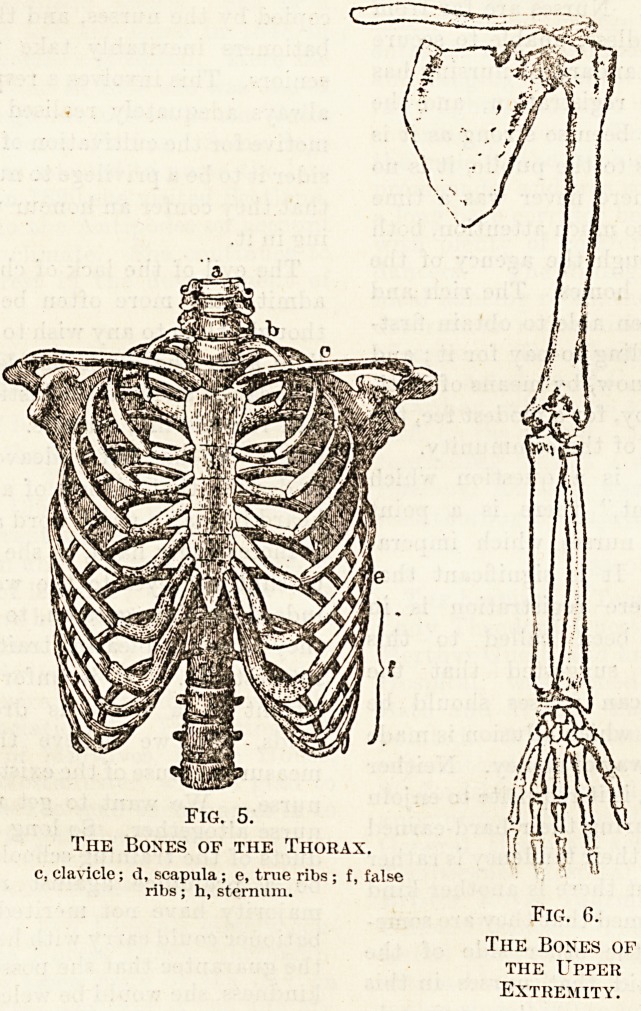


**Fig. 7. f3:**
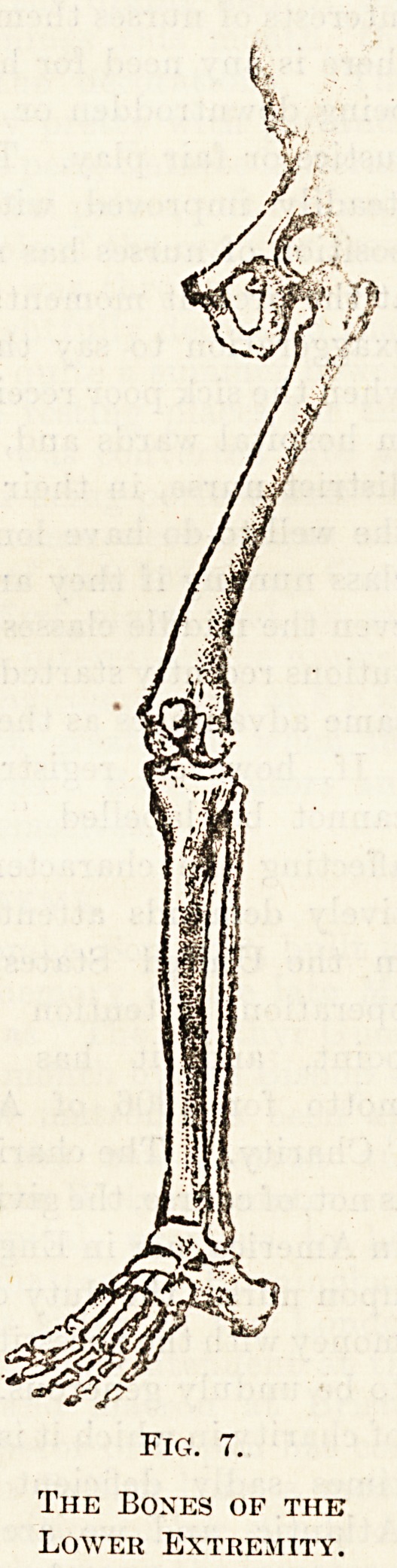


**Figure f4:**